# High-Performance p-Cu_2_O/n-β-Ga_2_O_3_ Heterojunction Barrier Schottky Diodes with Copper Contact

**DOI:** 10.3390/nano15241840

**Published:** 2025-12-05

**Authors:** Xiaohui Wang, Xuhui Liu, Mujun Li, Haozhe Yu, Kah Wee Ang, Chun Zhang Chen, Yue Geng, Qing Wang, Hongyu Yu

**Affiliations:** 1 Peng Cheng Laboratory, Shenzhen 518000, China; 2 School of Microelectronics, Southern University of Science and Technology, Shenzhen 518055, China; 3 Department of Electrical and Computer Engineering, National University of Singapore, Singapore 117583, Singapore; 4 School of Integrated Circuit, Shenzhen Polytechnic University, Shenzhen 518055, China

**Keywords:** β-Ga_2_O_3_, power electronics, JBS diode, low work function, Cu_2_O

## Abstract

This study demonstrates the fabrication of high-performance p-Cu_2_O/n-β-Ga_2_O_3_ heterojunction barrier Schottky (JBS) diodes using copper as a low-work-function anode metal. By optimizing the Cu_2_O spacing to 4 μm, the device achieves a turn-on voltage of 0.78 V, a breakdown voltage of 1700 V, and a specific on-resistance of 5.91 mΩ·cm^2^, yielding a power figure of merit of 0.49 GW/cm^2^. The JBS diode also exhibits stable electrical characteristics across the temperature range of 300–425 K. Under a 200 V reverse stress for 5000 s, the JBS diode shows only a 4.16% degradation in turn-on voltage and a 1.15-fold increase in dynamic specific on-resistance variation, highlighting its excellent resistance to stress-induced degradation. These results indicate that Cu_2_O/Ga_2_O_3_ JBS diodes are promising candidates for next-generation high-efficiency and high-voltage power electronic applications.

## 1. Introduction

The continuous growth in global electricity consumption has intensified environmental challenges, highlighting the urgent need to improve energy efficiency. In power electronic systems, reducing semiconductor device losses is crucial for enhancing efficiency and reliability [1,2]. Progress in this field relies on next-generation semiconductor materials that can simultaneously achieve low power consumption, high power density, and high-voltage capability, thereby enabling sustainable and high-performance energy solutions [3,4,5,6]. Over the past decade, beta-phase gallium oxide (β-Ga_2_O_3_) has attracted significant interest for high-performance power electronics owing to its wide bandgap (4.5–4.8 eV), high critical electric field (8 MV/cm), large Baliga’s figure of merit and superior radiation tolerance compared to GaN [7,8,9,10,11]. Moreover, the feasibility of cost-effective melt-grown single-crystal substrates further enhances its potential for commercial applications [12,13,14,15].

For Schottky barrier diodes (SBDs), which are widely employed in power applications, the dominant losses arise from conduction losses and leakage currents [16,17,18]. Two strategies have been proposed to mitigate these limitations, one of which is enlarging the device area to reduce the forward voltage drop. For example, Ampere-class Cu_2_O/Ga_2_O_3_ trench heterojunction barrier Schottky (JBS) diodes with an active area of 1.4 mm × 1.4 mm achieve a low turn-on voltage (V_ON_) of 1.1 V but suffer from a relatively low breakdown voltage (BV) of 986 V [19]. Similarly, NiO/Ga_2_O_3_ JBS diodes with areas of 9 mm^2^/16 mm^2^ achieve V_ON_ values of 0.94 V and 0.95 V, respectively, but their BVs remain limited to 550 V and 500 V [20]. Therefore, the simultaneous realization of JBS diodes with both low V_ON_ and high BV is of critical importance.

Most previously reported NiO/Ga_2_O_3_ JBS diodes employed high-barrier Schottky metals (e.g., Ni) to suppress reverse leakage [21,22,23,24,25]. However, due to insufficient electric field management, they typically exhibited a V_ON_ of ~1 V, thereby failing to fully exploit the advantages of the JBS structure. An alternative approach is to construct JBS diodes with low-work-function anode metals, which reduce the barrier height (Φ_bi_) and further lower the V_ON_. For instance, a Mo-anode JBS diode with a low V_ON_ of 0.64 V and a high BV of 2340 V has been reported [26]. Similarly, a TiN-anode JBS diode demonstrated an excellent V_ON_ of 0.52 V with a BV of 1150 V [27]. However, the trade-off between achieving low V_ON_ and high BV, together with the limited understanding of the long-term reliability of Cu_2_O/Ga_2_O_3_ JBS diodes, underscores the need for further investigation.

In this work, we present a Cu_2_O/Ga_2_O_3_ JBS structure incorporating a low-work-function copper (Cu) (~4.65 eV) anode to balance the low V_ON_ and high BV. The proposed JBS achieves a V_ON_ as low as 0.78 V and a power figure of merit (PFOM) of 0.49 GW/cm^2^, reflecting great potential for β-Ga_2_O_3_ power electronics. The device demonstrates stable electrical characteristics over a wide temperature range (300–425 K) and strong resistance to stress-induced degradation. These findings highlight a viable pathway for advancing β-Ga_2_O_3_-based power electronics with enhanced performance and long-term reliability.

## 2. Device Structure and Fabrication Process

**Figure 1:**
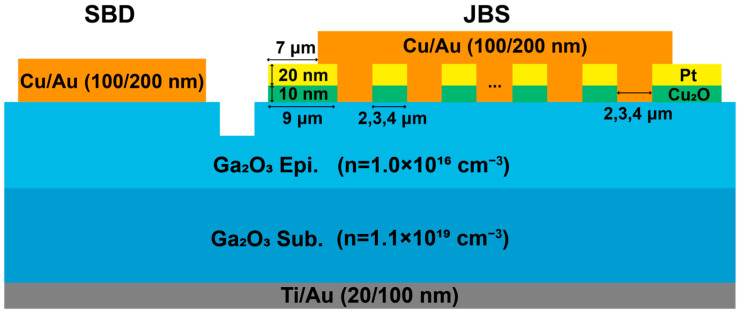
Cross-sectional schematic image of the β-Ga_2_O_3_ SBD and JBS diodes with Cu anode.

Figure 1 presents the β-Ga_2_O_3_ JBS diodes and SBD featuring a Cu anode. The β-Ga_2_O_3_ epitaxial wafer employed in this work was provided by Novel Crystal Technology (NCT), Japan. The wafer consists of a 10-μm Si-doped n-type drift layer with a concentration of 1.0 × 10^16^ cm^−3^, grown on a heavily Sn-doped bulk substrate (650-μm) with a doping concentration of 1.1 × 10^19^ cm^−3^. The fabrication process started from wafer cleaning in acetone, isopropyl alcohol, and deionized water. Mesa isolation was achieved by inductively coupled plasma (ICP) etching using BCl_3_ as the etchant and followed by a 10 min piranha solution treatment (H_2_SO_4_:H_2_O_2_ = 4:1) to remove surface impurities and organic residues. The wafer backside deposited a Ti/Au (20/100 nm) via electron beam evaporation (EBE) and annealed at 510 °C for 1 min in N_2_ ambient using rapid thermal annealing (RTA) to form ohmic contacts. Subsequently, RF magnetron sputtering at room temperature was employed to deposit the p-type Cu_2_O film on the β-Ga_2_O_3_, followed by a lift-off process. A Pt layer was then in situ deposited on the Cu_2_O to form p-type ohmic contact, followed by the deposition of a Cu/Au (100/200 nm) on the top of the Cu_2_O. For consistency, all the β-Ga_2_O_3_ diodes employed the same anode metal with a 50-μm radius to ensure comparable forward current levels. Moreover, in the JBS diode structure, the width of the p-Cu_2_O junction termination extension (JTE) region was designed to be 9 μm, while the width and spacing of the p-Cu_2_O rings were set to 2, 3, 4 μm.

The p-type Cu_2_O film was sputtered by RF magnetron sputtering using a high-purity Cu_2_O (99.99%) target. The sputtering was carried out at an RF power of 100 W for 3 min. During the deposition, flow rates of 50 sccm Ar and 2 sccm O_2_ were used to regulate the hole concentration within the Cu_2_O layers. The resulting p-Cu_2_O film exhibited a hole concentration of 2.7 × 10^19^ cm^−3^ and a mobility of 0.28 cm^2^/V∙s, as measured by room-temperature Hall effect measurements.

In this study, the hole concentration and mobility of the Cu_2_O films were characterized using an HL5500PC Hall effect measurement system. The surface morphology and root mean square (RMS) roughness were examined by non-contact 3D surface metrology and device inspection (Sensofar Tech, S.L. (Terrassa, Spain)). The scanning electron microscopy (SEM) image was obtained by EM-40, COXEM. Electrical characterization was carried out using a Keithley 4200-SCS parameter analyzer for room-temperature forward current-voltage (I–V), temperature-dependent I–V, and stress time-dependent I–V measurements, as it provides high-resolution, low-voltage capability suitable for forward characterization. In contrast, the reverse characteristics were measured with an Agilent B1505A system, which can supply the kilovolt-level reverse bias required for high-voltage β-Ga_2_O_3_ devices.

## 3. Results and Discussion

**Figure 2:**
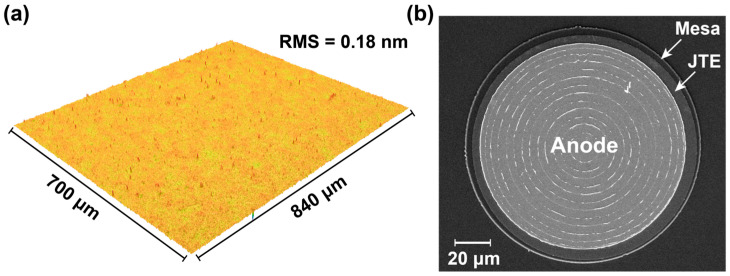
(**a**) The non-contact 3D surface profiler image of Cu_2_O film. (**b**) The SEM image of the fabricated JBS diode with a spacing of 4 μm.

Figure 2 presents the surface morphology and SEM image of the Cu_2_O/Ga_2_O_3_ JBS diode. Figure 2a shows the surface morphology of the Cu_2_O film, measured using a non-contact 3D surface metrology and device inspection system over a scanning area of 700 μm × 840 μm. The root mean square (RMS) roughness is determined to be 0.18 nm, demonstrating an exceptionally smooth and uniform Cu_2_O surface. Such low roughness is highly beneficial for device fabrication, as it suppresses interface defect formation at the Cu_2_O/Ga_2_O_3_ heterojunction. Figure 2b displays the SEM image of the fabricated JBS diode, revealing a circular anode pattern with well-defined concentric ring structures. These rings represent the characteristic architecture of the JBS diode design, which facilitates efficient current spreading and a uniform electric field distribution within the active region. The device diameter and fine structural details are distinctly resolved, with a scale bar of 20 μm.

**Figure 3:**
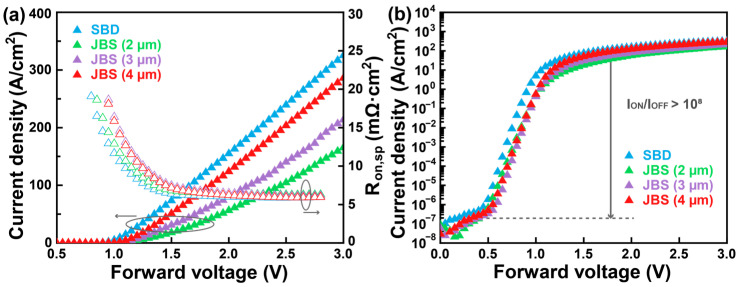
(**a**) Linear I–V characteristics and the extracted differential R_on,sp_ as a function of forward bias for comparison between the β-Ga_2_O_3_ JBS diodes and SBD. (**b**) Semi-logarithmic I–V characteristics for these devices.

Figure 3a illustrates the forward I–V characteristics of the fabricated β-Ga_2_O_3_ JBS diodes with different Cu_2_O spacing values (2, 3, and 4 μm), along with a β-Ga_2_O_3_ SBD for comparison. The ideality factors of the β-Ga_2_O_3_ JBS diodes extracted from the 0.6–1.0 V region are 1.19, 1.32, and 1.21 for spacings of 2, 3, 4 μm, respectively, while the β-Ga_2_O_3_ SBD exhibits an ideality factor of 1.16. These values deviate slightly from unity, indicating that Shockley–Read–Hall (SRH) recombination at the heterojunction interface is the dominant transport mechanism under the forward bias, particularly at lower voltages. The relatively higher ideality factor observed in the JBS diode with 3 μm spacing implies enhanced recombination activity, which may arise from localized interface states or inhomogeneous barrier formation [28,29]. As the spacing of the Cu_2_O region rises, the forward current density correspondingly increases. This behavior can be attributed to the enlarged conduction path in devices with larger spacing, which reduces the influence of the Schottky barrier regions and allows more carriers to flow through the p–n heterojunction. Consequently, the extracted differential specific on-resistance (R_on,sp_) of JBS diodes decreases from 6.27 mΩ·cm^2^ to 6.12 mΩ·cm^2^ and 5.91 mΩ·cm^2^ as the spacing increases from 2 to 3 and 4 μm, respectively. The reduction in R_on,sp_ reflects the improved carrier transport efficiency enabled by the larger effective junction area, which lowers the contribution of series resistance from the depletion region [30]. For comparison, the β-Ga_2_O_3_ SBD exhibits a slightly lower R_on,sp_ of 5.86 mΩ·cm^2^, highlighting the high-quality interface between the Cu anode and β-Ga_2_O_3_.

Figure 3b depicts the semi-logarithmic I–V characteristics of the β-Ga_2_O_3_ JBS diodes and SBD. For a fair comparison, the current density of the SBD and JBS diodes was calculated by normalized the measure current to the full anode area. All diodes exhibit excellent rectifying behavior, with I_ON_/I_OFF_ ratios exceeding 10^9^, confirming the effective suppression of reverse leakage current and the strong rectification capability of the heterojunction structures. The V_ON_, defined at current density of 1 A/cm^2^, was extracted to be 0.68 V for the SBD and 0.79 V, 0.78 V, and 0.78 V for the JBS diodes with Cu_2_O spacings of 2, 3, and 4 μm, respectively. The slightly delayed turn-on behavior observed in the JBS diodes compared with the SBD originates from the additional potential barrier introduced by the p–n heterojunctions. This delayed conduction is an intrinsic feature of JBS structures, where the p–n heterojunction regions are designed to block leakage under reverse bias while the Schottky regions dominate forward conduction [31,32].

**Figure 4:**
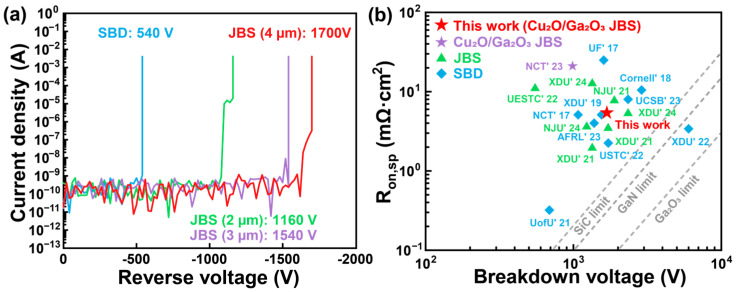
(**a**) Reverse I–V characteristics of the β-Ga_2_O_3_ JBS diodes with different Cu_2_O spacing values (2, 3, and 4 μm) and SBD. (**b**) Benchmark plot of the reported state-of-the-art works on β-Ga_2_O_3_ diodes.

The reverse I–V characteristics of the diodes are summarized in Figure 4a, with the cathode grounded and the anode reversely biased from 0 V until the reverse current reaches the breakdown criterion of 0.01 A. The SBD shows a BV of 540 V, whereas the JBS diodes achieve significantly higher BV values of 1160 V, 1540 V, and 1700 V for spacings of 2, 3, and 4 μm, respectively. In the JBS diodes, the reverse current shows a relatively weak dependence on the applied reverse bias, primarily due to the depletion-induced pinch-off at the Cu_2_O/Ga_2_O_3_ p–n heterojunctions [33]. Moreover, a wider p-region spacing allows the depletion layer to extend more fully, resulting in a more uniform electric field distribution, alleviating local field crowding, and thereby improving the breakdown characteristics of the device. By contrast, the SBD displays a sudden increase in reverse current at low reverse voltage, which may originate from the impact of interface barrier. These reverse I–V characteristics clearly highlight the superiority of the JBS diodes over the SBD. Figure 4b presents the benchmark plot of the R_on,sp_ versus BV for the state-of-the-art β-Ga_2_O_3_ diodes. The fabricated JBS diode with a 4 μm spacing achieves a PFOM value of 0.49 GW/cm^2^. This performance is comparable to that of the widely investigated NiO/Ga_2_O_3_ JBS diodes, highlighting the strong promise for use in next-generation high-performance power-electronics technology. Table 1 summarized the electrical parameters of all β-Ga_2_O_3_ diodes. The SBD exhibits a low R_on,sp_ of 5.86 mΩ·cm^2^ and BV of 540 V, corresponding to a PFOM value of 0.05 GW/cm^2^. In comparison, the JBS diodes with spacings of 2 μm and 3 μm achieve PFOM values of 0.21 GW/cm^2^ and 0.39 GW/cm^2^, respectively. Notably, the JBS diode with a 4 μm spacing demonstrates a low R_on,sp_ of 5.91 mΩ·cm^2^ and a high BV of 1700 V, further yielding a PFOM value of 0.49 GW/cm^2^.

**Figure 5:**
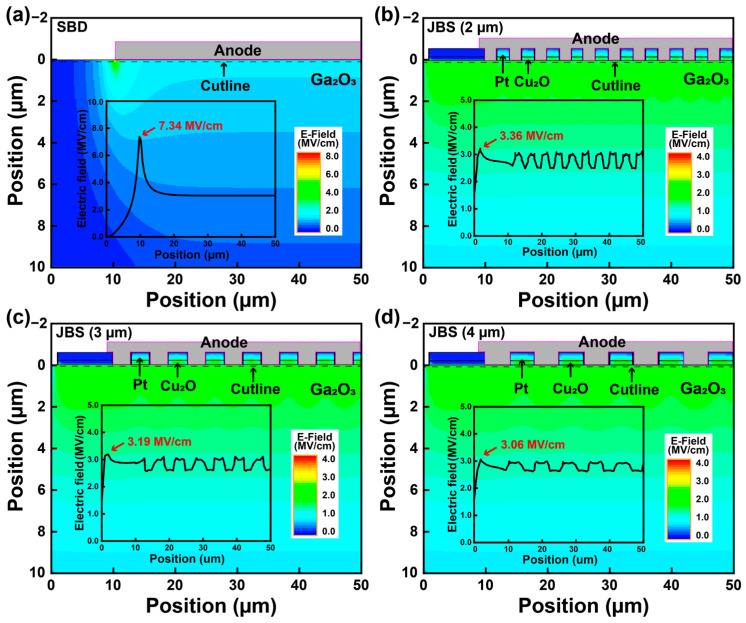
Electric field distribution of the (**a**) β-Ga_2_O_3_ SBD and (**b**–**d**) β-Ga_2_O_3_ JBS diodes with different Cu_2_O spacing values (2, 3, and 4 μm).

To further elucidate the influence of Cu_2_O spacing on the breakdown performance, electric field simulations were conducted on these devices under a reverse voltage of 1000 V. Figure 5 compares the electric field distributions of the β-Ga_2_O_3_ SBD and the JBS diodes with Cu_2_O spacings of 2, 3, and 4 μm, respectively. As shown in Figure 5a, the SBD exhibits pronounced field crowding at the Schottky edge, where the peak electric field reaches 7.34 MV/cm, indicating that the breakdown is dominated by anode edge localization [34]. In contrast, the β-Ga_2_O_3_ JBS diodes demonstrate significantly suppressed electric fields of 3.36 MV/cm, 3.19 MV/cm and 3.06 MV/cm for Cu_2_O spacings of 2, 3, and 4 μm, respectively, accompanied by a smoother and more uniformly distributed field profile along the cutline. The periodic p-type Cu_2_O regions redistributes the reverse-bias space charge, creating multiple depletion junctions that spread the electric field within the β-Ga_2_O_3_ drift layer [35,36]. This effect effectively mitigates edge crowding and yields a more uniform field profile. Consequently, the JBS diodes with Cu_2_O spacings substantially improves electric field modulation, consistent with the experimentally observed enhancement in breakdown capability.

**Figure 6:**
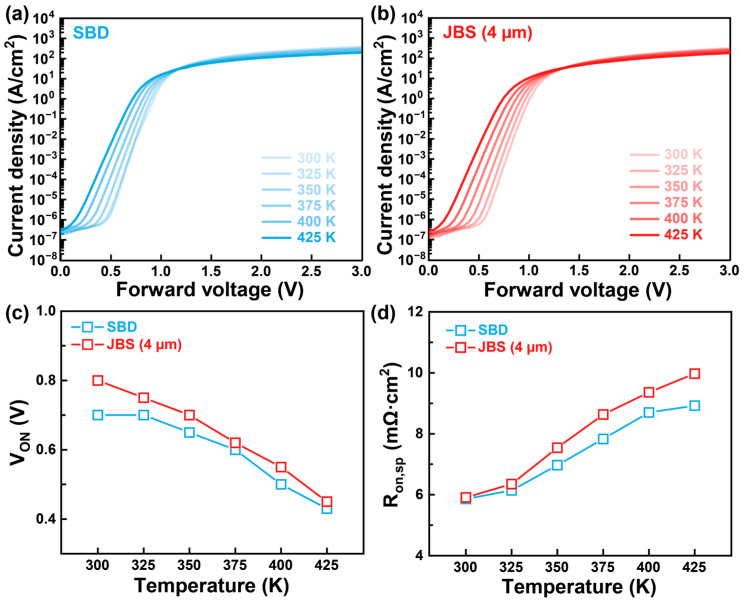
Temperature-dependent I–V characteristics of β-Ga_2_O_3_ (**a**) SBD and (**b**) JBS diode. (**c**,**d**) are the extracted V_ON_ and R_on,sp_ parameters of the β-Ga_2_O_3_ JBS diode and SBD.

Based on the aforementioned electrical characteristics, the JBS diode with a spacing of 4 μm demonstrates superior performance. Therefore, the subsequent evaluations primarily focus on a comparative analysis between the 4 μm JBS diode and the reference SBD. To investigate the thermal stability of these devices, the electrical performance was characterized over the temperature range from 300 K to 425 K. Figure 6 shows the temperature-dependent electrical characteristics of the fabricated β-Ga_2_O_3_ JBS diode (4 μm) and SBD. Figure 6a,b present the forward I–V characteristics of the SBD and JBS, respectively. Both devices exhibit typical rectifying behavior, and the V_ON_ decreases gradually with increasing temperature, which can be explained by enhanced thermionic emission and the temperature dependence of the Φ_bi_. As temperature rises, the increased carrier energy facilitates barrier crossing, resulting in a lower V_ON_. Figure 6c,d summarize the extracted parameters as a function of temperature. As shown in Figure 6c, the V_ON_ decreases from 0.78 V to 0.45 V for the JBS diode and from 0.68 V to 0.43 V for the SBD as the temperature increases. This reduction can be attributed to reduced trap-assisted transport at elevated temperatures, which improves the effective interface quality and reduces barrier inhomogeneity. Figure 6d depicts the temperature dependence of the R_on,sp_. With increasing temperature, the R_on,sp_ increases to 9.97 mΩ·cm^2^ for the JBS diode and 8.92 mΩ·cm^2^ for the SBD at 425 K. This increase primarily due to enhanced phonon scattering in the drift layer, which lowers carrier mobility and thereby increases conduction resistance. Notably, the JBS diode consistently exhibits a higher V_ON_ and R_on,sp_ compared to the SBD, which can be attributed to the p–n heterojunctions in the JBS structure. These heterojunctions improve junction uniformity and suppress leakage, but at the cost of increased conduction path resistance [37,38].

**Figure 7:**
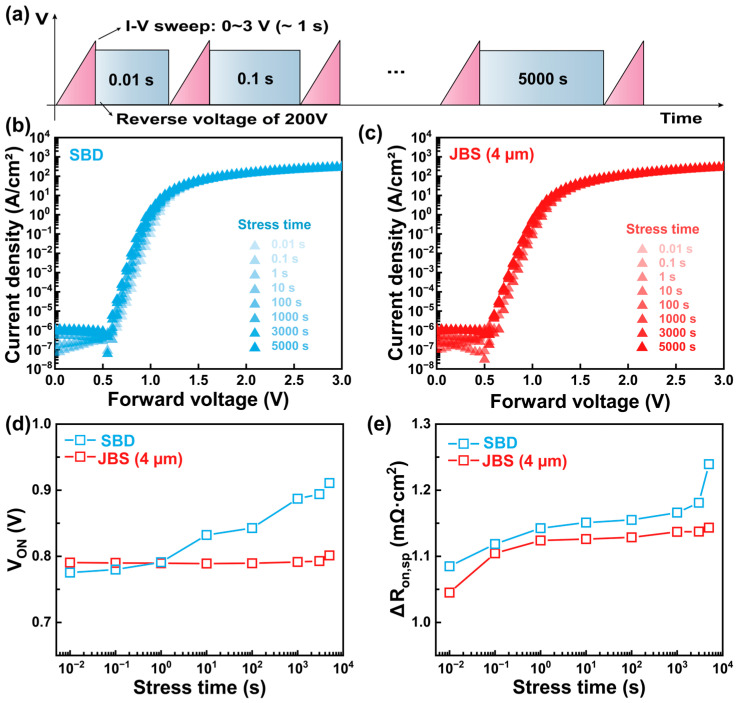
(**a**) Measurement-stress-measurement scheme for long-term reliability test. Linear-scale forward I–V characteristics of the β-Ga_2_O_3_ (**b**) SBD and (**c**) JBS diode after various stress times. (**d**,**e**) are the extracted corresponding V_ON_ and ∆R_on,sp_ as a function of the stress time, respectively.

Figure 7 illustrates the forward electrical characteristics of the β-Ga_2_O_3_ SBD and JBS (4 μm) diodes under different stress time at room temperature. Figure 7a illustrates the measurement–stress–measurement scheme used to evaluate the long-term reliability of the SBD and JBS devices. Figure 7b,c present the forward I–V curves after stress times ranging from 0.01 s to 5000 s under reverse stress of 200 V, a level selected to prevent premature breakdown during extended stress testing. Both devices exhibit a gradual increase in forward current with prolonged stress time, which can be ascribed to stress-induced barrier modification and the generation of interface state. These defect states locally reduce the effective Φ_bi_, thereby enhancing carrier transport [39,40]. The degradation effect is more evident in the SBD, whereas the JBS diode exhibits improved stability because the p–n heterojunctions effectively suppress barrier fluctuations and limit the influence of localized interface degradation [41,42]. Figure 7d,e summarize the extracted electrical parameters as a function of stress time. As shown in Figure 7d, the V_ON_ of the SBD increases significantly with prolonged stress time, deteriorating by 17.45%. This behavior arises from charge trapping and barrier inhomogeneity, which hinder uniform current conduction [43]. In contrast, the JBS diode shows only a 4.16% deterioration in V_ON_, indicating that the p–n heterojunctions stabilize the barrier profile and ensure more uniform potential distribution [44]. Similarly, the dynamic specific on-resistance variation (∆R_on,sp_) increases with stress time for both devices due to enhanced phonon scattering and the accumulation of trapped charges. However, the JBS diode consistently maintains a lower ∆R_on,sp_, increasing only to 1.15 after 5000 s, compared to 1.24 for the SBD. This reduced degradation highlights the capability of the JBS diode to alleviate current crowding and suppress defect-assisted conduction pathways [45]. Overall, these results confirm that the JBS structure offers enhanced reliability and stronger resistance to stress-induced performance degradation compared with the conventional SBD.

## 4. Conclusions

In summary, the Cu_2_O/Ga_2_O_3_ JBS diode achieves a favorable balance between low conduction loss and high BV, benefiting from the combined roles of the Cu anode and the p–n heterojunctions. The JBS diode with a 4 μm spacing features a low V_ON_ of 0.78 V, a high BV of 1700 V and a R_on,sp_ of 5.91 mΩ·cm^2^, yielding a PFOM of 0.49 GW/cm^2^. Moreover, it also exhibits excellent thermal stability and superior resistance to stress-induced degradation, with only a 4.16% increase in V_ON_ and a modest 1.15-fold rise in ∆R_on,sp_ under a 200 V reverse stress for 5000 s. These findings validate the effectiveness of the Cu_2_O/Ga_2_O_3_ JBS structure as a robust strategy for advancing β-Ga_2_O_3_-based power electronics, paving the way toward the next generation of high-efficiency and high-voltage devices.

## Figures and Tables

**Table 1:** Summary of R_on,sp_ and BV for the β-Ga_2_O_3_ diodes.

Samples	R_on,sp_ (mΩ·cm^2^)	BV (V)	PFOM (GW/cm^2^)
SBD	5.86	540	0.05
JBS (2 μm)	6.27	1160	0.21
JBS (3 μm)	6.12	1540	0.39
JBS (4 μm)	5.91	1700	0.49

## Data Availability

The data that support the findings of this study are available from the corresponding authors upon reasonable request.
